# Draft Genome of *Acidovorax kalamii* Strain JM16, Isolated from Skin Mucus of Zebrafish (Danio rerio)

**DOI:** 10.1128/mra.00667-22

**Published:** 2022-10-17

**Authors:** Jacquelin Machado-Conteras, Kayla Thomas, Anna K. S. Jozwick

**Affiliations:** a Center for Natural Sciences, Goucher College, Baltimore, Maryland, USA; University of Southern California

## Abstract

Acidovorax kalamii strain JM16 was isolated from the skin mucus of the zebrafish, Danio rerio. Its genome is 5.3 Mb with a 65.6% GC content and encodes quorum sensing capabilities, which could contribute to ecosystem functioning within the fish host skin bacterial community.

## ANNOUNCEMENT

Acidovorax kalamii strain JM16 is a Gram-negative, aerobic *Betaproteobacteria* member isolated from the skin mucus of zebrafish, Danio rerio. Members of this bacterial genus have been classified as environmental microbes, endosymbionts of earthworms, phytopathogens, and opportunistic human pathogens (1–9). *A. kalamii* was first described in 2018 (10) and cultured from a water sample of the River Ganges in India, yet relationships or ecosystem roles are undescribed.

*Acidovorax kalamii* strain JM16 was cultured from swabs of the skin of healthy adult zebrafish, Danio rerio (11), under a protocol approved by Goucher College’s IACUC. Serial dilutions were grown on R2A medium for 96 h. The culture was then purify-streaked three successive times on tryptic soy agar (TSA) with an incubation at 28°C for 48 h each and immediately glycerol stocked. DNA extraction and PCR amplification of ~1,465 bp of 16S rRNA genes were performed as described in the protocol at https://doi.org/10.6084/m9.figshare.21203108. Genewiz (Plainfield, NJ) performed Sanger sequencing on purified PCR products. The 16S rRNA gene sequences were trimmed manually using SnapGene Viewer (version 5.2.2; GSL Biotech LLC, San Diego, CA). NCBI BLASTn database comparison identified Acidovorax delafieldii (NCBI accession number NR_028714) as the best BLAST match. *A. kalamii* strain JM16 was the only *Acidovorax* sp. isolated from the zebrafish skin mucus microbiome.

*A. kalamii* strain JM16 was purify-streaked from glycerol stock onto TSA, grown for 48 h at 28°C, and shipped to the Microbial Genome Sequencing Center (MiGS) in Pittsburgh, PA, for genome sequencing. DNA extracted using a Zymo DNA Miniprep with bead beating lysis (Zymo Research, Tustin, CA) was made in a library using an Illumina DNA prep kit with IDT 10-bp unique dual indices (UDI) and sequenced on an Illumina NextSeq 2000. A total of 5,995,914 paired-end 151-bp reads from 887,353,491 bp of raw sequence data was generated. MiGS provided demultiplexed, adapter sequence-trimmed reads, which we further processed in KBase.us Narrative Interface. Default parameters were used for all software, unless otherwise specified. Trimmomatic v0.36 (12) set to a head and post tail crop length of 15 and 150, respectively, leading and trailing minimum quality of 5, and minimum length of 130 bp produced good-quality reads as assessed using FastQC v.0.11.9 (13). SPAdes v3.15.3 (14) and Velvet v.1.2.10 (15) assemblies were performed. Quast v4.4 (16, 17) identified the SPAdes assembly of higher quality, with 76 contigs having an *N_50_* value of 149,384, GC content of 65.59%, and 167× coverage. The NCBI Prokaryotic Genome Annotation Pipeline (PGAP) (18–20) identified 4,813 coding genes, 1 complete rRNA gene, and 48 tRNAs. CheckM v1.0.18 (14) determined that the 5,297,352-bp genome was 99.12% complete with 0.4% contamination. GTDB-Tk0v1.7.0 (21) classified this isolate as *Acidovorax kalamii*, a different species than the best BLAST match (*A. delafieldii*).

**FIG 1:**
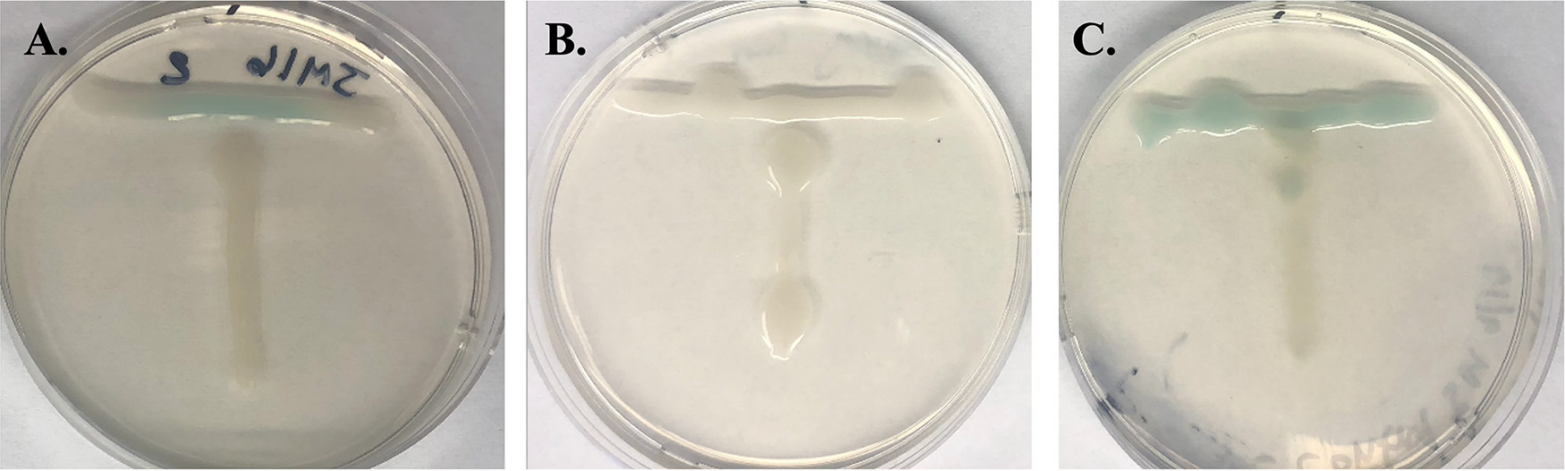
Representative images of the T-streak bioindicator assay for quorum sensing autoinducer 1 production. Horizontal growth of the bioindicator, Agrobacterium tumefaciens NTL4 pZLR4, will turn blue if AHL autoinducers from bacteria of interest are produced. Vertical growth is *Acidovorax kalamii* strain JM16 (A), the bioindicator itself (negative control) (B), and Yersinia ruckeri CSF007-82 (positive control) (C). Strains were streaked from single colonies on one-half TSA medium plus 75 μg/mL 5-bromo-4-chloro-3-indolyl-β-d-galactopyranoside (X-Gal) and photographed after 24 h of growth at 28°C.

*A. kalamii* strain JM16 (NCBI accession number OP171902) was a quorum sensing autoinducer 1 (acylhomoserine lactone [AHL]) producer (Fig. 1) isolated from the aerobic bacterial community of zebrafish skin mucus. All skin isolates were screened for AHL production using the bioindicator Agrobacterium tumefaciens NTL4 pZLR4 (22, 23). The genome of *A. kalamii* strain JM16 encoded an AHL quorum sensing system, including a LuxR family acylhomoserine lactone-binding transcriptional activator and *N*-acyl-l-homoserine lactone synthetase RhlL, supporting the culturing observations.

### Data availability.

This genome is part of BioProject PRJNA849171 and is BioSample SAMN29042293 with sequence reads (SRA accession number SRX15699340) and assembly (genome accession number JAMXHW010000000).
